# Transplantation of Deprenyl-Induced Tyrosine Hydroxylase-Positive Cells Improves 6-OHDA-Lesion Rat Model of Parkinson’s Disease: Behavioral and Immunohistochemical Evaluation

**Published:** 2013-05-05

**Authors:** Maryam Haji Ghasem Kashani, Mohammad Taghi Ghorbanian, Leili Hosseinpour

**Affiliations:** 1 Department of Biology, School of Biology, Damghan University, Damghan, Iran; 2 Institute of Biological Sciences, Damghan University, Damghan, Iran

**Keywords:** Bone Marrow Stromal Cells, Deprenyl, Cell Therapy, Parkinson’s Disease, 6-Hydroxydopamine

## Abstract

**Objective:**

There is longstanding experimental and clinical evidence that supports the idea that replacement of dopaminergic (DAergic) neurons can ameliorate functional disabilities of Parkinson’s disease (PD). The purpose of the present study is to examine the efficacy of transplantation of rat bone marrow stromal cell (BMSCs)-derived tyrosine hydroxylase-positive (TH^+^) cells induced by deprenyl into 6-hydroxydopamine (6-OHDA)-lesioned rat models, using behavioral tests and immunohistochemical evaluations.

**Materials and Methods:**

In this experimental study, undifferentiated BrdU-labeled BMSCs were incubated in serum-free medium that contained 10^-8^ M deprenyl for 24 hours. Afterwards, BMSCs were cultured for 48 hours in α-minimal essential medium (α-MEM) supplemented with 10% FBS, then differentiated into TH^+^ neurons. We randomly divided 24 hemiparkinsonian rats as follows: group 1 (control) received only medium, while groups 2 and 3 were injected with 2×10^5^ BMSCs and deprenyl-treated cells in 4 µl medium. Injections were made into the injured strata of the rats. Rotational behavior in response to apomorphine was tested before transplantation and at 2, 4, and 6 weeks post-graft. Animals were then sacrificed, and the brains were extracted for immunohistochemical and electron microscopic studies.

**Results:**

Apomorphine-induced rotation analysis indicated that animals with grafted cells in groups 2 and 3 exhibited significantly less rotational behavior than those in the control group at 2, 4, and 6 weeks after transplantation. Immunohistochemical analysis demonstrated that BrdU-labeled cells expressed specific neuronal markers, such as NF 200 and TH, at the implantation site. The presence of TH^+^ cells in conjunction with the reduction in rotation might show the capacity of grafted cells to release dopamine. Ultrastructural analysis revealed the presence of immature neurons and astrocyte-like cells at the graft site.

**Conclusion:**

TH^+^ neurons induced by deprenyl can be considered as a cell source for PD autograft therapy.

## Introduction

A number of psychiatric and movement disorders in humans, such as Parkinson’s disease (PD), are caused by the degeneration or dysfunction of dopaminergic (DAergic) neurons in the substantia nigra pars compacta with a subsequent reduction in striatal dopamine, which leads to a disruption in the normal function of the basal ganglia (1, 2). This highlights the importance of DAergic neurons for maintenance of health and explains why these cell types are the subject of extensive investigation. Clinical experiments using cell transplantation as a therapy for PD have been conducted since 1980.

The replacement of degenerated neurons with effectively functional exogenous potent stem cells is a more promising technique for tissue repair and regeneration in cases of PD (2). Fetal DAergic cells originate in the ventral mesencephalic tissue that has been obtained from fetuses, and have the potential to improve motor function in animal models of PD. However, technical difficulties in obtaining sufficient graft tissues, ethical considerations, and rejection of cells are limitations for the application of this therapy (3-5).

Over the last 20 years scientists have been searching for other reliable sources of midbrain DA neurons, of which stem cells appear to be promising candidates. There are three sources of stem cells that are currently being researched: embryonic stem cells (ESCs), neural stem cells (NSCs), and mesenchymal stem cells (MSCs). NSCs are isolated from embryonic or adult brains; they can only generate nervous system cell lines, and have less potential than ES cells (6, 7).

The ethical debate on using aborted embryo cells and the risk of tumor formation have limited the use of ESCs. Bone-marrow mesenchymal stem cells (BMSCs) can represent an alternative source of stem cells for cell replacement therapies (8-11). They are easily isolated from hematopoietic stem cells, avoid ethical problems, and can be used for autologous transplantation (12). Recently, many protocols that use *in vitro* differentiation of BMSCs into DAergic neurons have been developed (13, 14). These protocols involve the direct administration of growth factors and/or chemicals in cultures, or ectopic expression of their coding sequences within cells (15-17).

Previous studies have shown that neural-like cells can be produced *in vitro* from BMSCs following induction of deprenyl (18, 19). Selegiline, or L-deprenyl, is a monoamine oxidase- B (MAO-B) inhibitor that slows the progression of PD. Deprenyl is known to increase the survival of cultured nigral DAergic neurons, protecting them from oxidative stress. The trophic effects of selegiline may play a significant role in the treatment of neurodegenerative diseases (20, 21).

It has been reported that deprenyl can protect hippocampal neurons from excitotoxic damages, most likely by induction of NGF protein. Studies also show that it may have the neuroprotective effects and trophic effects of deprenyl (22, 23). In this study, we have generated TH^+^ cells from rat BMSCs by induction of deprenyl, as previously reported, and then transplanted them into 6-hydroxydopamine (6-OHDA)-treated rats in order to investigate the clinical efficacy of these cells.

## Materials and Methods

### Animals

The experimental protocol was approved by the Research and Ethics Committee of Damghan University. Adult male Sprague-Dawley rats that weighed 200-250 g were purchased from Razi Institute, Karaj, Iran. Animals were kept under standard laboratory conditions with a 12 hours light/dark cycle and ad libitum food and water throughout the experiments.

### Preparation of bone marrow stromal cells and production of tyrosine hydroxylase–positive neurons

Animals were killed and femurs were dissected out. The marrow was extruded with α-minimal essential medium (α-MEM). The extracted solution was centrifuged at 150 x g for 10 minutes after which the cell pellet was resuspended in α-MEM supplemented with 10% FBS, penicillin (100 µM/ml, Gibco, USA), and streptomycin (100 µM/ml; Gibco) in a 25 cm2 tissue culture flask at 37˚C and 5% CO_2_ accord ing to a protocol by Rismanchi et al. (24). The culture medium was changed every 3-4 days to remove any non-adherent cells. When the flask reached 80% confluency (usually within two weeks), cells were harvested by incubation in 0.25% trypsin and 0.5 mM EDTA (Merck, Germany) at 37˚C for 3-4 minutes, after which they were subcultured.

Continuous subculturing of cells was performed for five passages. The fifth passage of the cells were treated with serum-free medium (25, 26) that contained 10^-8^ M of deprenyl for 24 hours, then cultured in α-MEM that contained 10% FBS for 48 hours (23, 24).

### Identification of transplanted cells

NF-200, synapsin, and TH immunocytochemistry were used to identify differentiated cells (19). Cells were cultured on gelatinized coverslips and fixed in 4% paraformaldehyde for 20 minutes at 4˚C, then permeabilized in 0.1% triton X-100 for 15 minutes and blocked in 10% normal goat serum for 15 minutes. Cells were incubated with primary antibodies, mouse anti-NF200 (Sigma, N5389, Germany) and rabbit anti-TH (Chemicon, AB152, USA), overnight at 4˚C. After three washes in 0.01 M phosphate buffered saline (PBS), the cells were incubated with secondary antibodies at 37˚C for 30 minutes. The secondary antibodies were HRP and FITC-conjugated anti-rabbit for the TH marker and FITC-conjugated anti-mouse for NF-200 (27). Expressions of TH and Nurr1 were determined by reverse transcription-polymerase chain reaction (RTPCR). Total RNA was extracted using a CinnaGen kit and 0.5 µg of total RNA was transcribed into cDNA with a Fermentase-k1622 kit. Subsequent PCR was performed with 5 µg of synthesized cDNA, 1×PCR buffer, 50 mM of MgCL2, 10 mM of dNTPs, 10 pmol of forward and reverse primers, 0.25 µl Taq DNA polymerase enzyme, and injection water with a terminal volume of 25 µl in thermal cycler with 34 cycles.

Table 1 lists the primers used in this study. β_2_ microglobulin (β_2_M) was used as the housekeeping (internal control) gene. RT-PCR was carried out in a master cycler (Eppendorf, Germany) using the following cycling parameters: initial denaturation at 94˚C for 2 minutes; followed by 34 cycles according to the program; denaturation at 94˚C for 30 seconds; annealing at 55˚C for 30 seconds; extension (elongation) at 72˚C for 30 seconds; at the end of 34 th cycle, the end stage of terminal extension was carried out at 72˚C for 5 minutes. The experiment was repeated three times for each sample; all stages were controlled in the cDNA preparation stage by deleting the RNA sample and reverse transcriptase, and in the RT-PCR stage by deleting Taq polymerase and the cDNA product.

### Unilateral corpus striatum 6-OHDA lesions and transplantation

Adult male Sprague-Dawley rats (weights: 250-300 g) received stereotaxic injections of 3 µl 6-OHDA (10 µg/µl dissolved in 1% ascorbate- saline) into their left striata via a metal cannula attached to a 10 µl Hamilton microsyringe at a rate of 1 µl/min. The injection site (AP + 1 mm, L-3 mm, V + 5 mm from the bregma) was according to the Paxinos and Watson atlas (29). After two weeks, lesioned animals were selected for transplantation surgery by counting apomorphine (0.05 mg/kg i.p.) -induced turning behavior (28-30); only animals that showed at least seven full turns/min against the lesioned side were selected for transplantation surgery (31). The rotation tests were repeated at 2, 4, and 6 weeks after transplantation.

To harvest donor cells for transplantation experiments, BrdU labeled deprenyl-treated cells (3) were incubated with 0.25% trypsin/0.04% EDTA at room temperature for 5 minutes. Cell suspension was then prepared at a concentration of 2×10^5^ cells in 5 µl medium. The cell viability was ~90% just prior to grafting. The cell suspension was injected into the injured striatum of each rat by using a stereotaxic apparatus and automatic microinjection pump at an injection rate of 1 µl/min (32).

Hemiparkinsonian rats were randomly divided into three groups: i. those that received only medium (control), ii. those transplanted with BMSCs, and iii. those that received an injection of deprenyl-treated cells.

**Table 1:** The primers used for reverse transcription-polymerase chain reaction analysis


Gene	Predicted size	Primer sequence	Accession number

**B2M**	318 bp	F: 5'-CCG TGA TCT TTC TGG TGC TT-3'	NM-012512
		R: 5'-TTT TGG GCT TCA GAG TG-3'	
**Nurrl**	638 bp	F: 5 ׳-TCC CGG AGG AAC TGC ACT TCG-3'	U-72345
		R: 5 ׳-GTG TCT TCC TCT GCT CGA TCA-3'	
**TH**	276 bp	F: 5'-TGT CAC GTC CCC AAG GTT CAT -3'	NM-012740
		R: 5'–CGT GGG ACC AAT GTC TTC AGT G- 3'	


### Immunohistochemical study

Identification of BrdU-labeled cells was performed via immunocytochemical staining. Six weeks after implantation, animals were anesthetized and transcardially perfused. Rats' brains were postfixed with 4% paraformaldehyde, processed for paraffin embedding, and sectioned into 5 µm thicknesses.

A series of sections were prepared for BrdU, TH, and NF-200 immunostaining. A number of the paraformaldehyde-fixed brains were incubated in 30% sucrose until they sank. Sections were cut on a freezing microtome at 40 µm, and processed for TH and BrdU immunohistochemistry.

The sections were rehydrated, incubated in 50% formamide/2X standard sodium citrate (SSC: 0.3 M NACl, 0.03 M sodium citrate) for 120 minutes at 60˚C, washed with 2X SSC for 10 minutes (both at room temperature), incubated in 2N HCl at 37˚C for 30 minutes, rinsed in 0.1 boric acid (pH=8.5) for 10 minutes, and washed in PBS. The sections were then incubated in blocking serum, followed by incubation with mouse anti-BrdU monoclonal antibody (Sigma, St. Louis, MO, USA, B2531) or anti- TH antibody overnight at 4˚C, and followed by labeling with anti-mouse IgG-peroxidase antibody in goat (Sigma, A9917, USA) for 2 hours at room temperature (33).

For double immunostaining, some sections were incubated with mouse anti-BrdU monoclonal antibody and subsequently with secondary antibody conjugated with rhodamine, while the secondary antibody for anti-NF200 or anti- TH antibodies was conjugated with FITC. Finally the two images were merged.

### Statistical analysis

Statistical analyses were carried out using one-way ANOVA with Tukey’s multiple comparison. For each parameter, the significance level was determined using SPSS (version 16). Data are expressed as mean ± SEM. P≤0.05 was considered statistically significant.

## Results

### Characterization of transplanted cells

**Fig 1:**
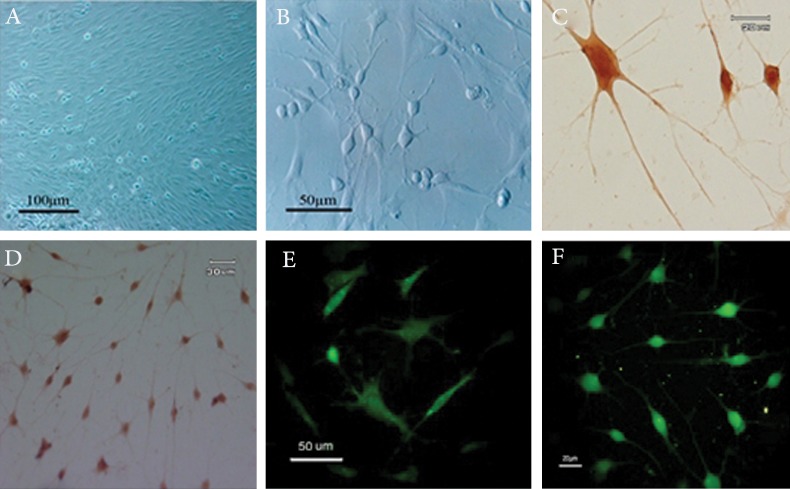
Deprenyl-treated rat mesenchymal stem cells (MSCs) display neural morphology and express neuronal markers. A. Fifth passage of cultured bone marrow MSCs (BMSCs); B cultured BMSCs following neuronal induction by deprenyl; C, D. treated cells demonstrated immunoreactivity for tyrosine hydroxylase (TH); E. synaptophysin; and F. NF200.

Cultured BMSCs with spindle-shaped morphology and deprenyl-treated BMSCs with neural morphological characteristics are shown in Figs 1 A and B. In addition, immunocytochemical evaluations in figure 1 C-F show that the majority of treated cells in the microscopic field were immunopositive for TH, NF200, and synapsin.

**Fig 2:**
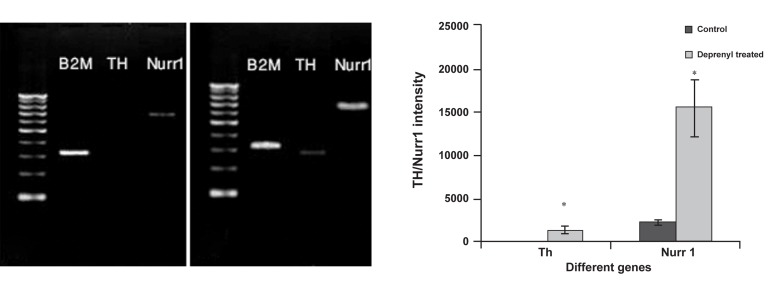
RT-PCR analysis of tyrosine hydroxylase (TH) and Nurr1 expression in deprenyl-treated and untreated rat bone marrow mesenchymal stem cells (BMSCs) . A. There was no mRNA expression of TH and a weak band of Nurr1 in untreated cells; B. mRNA expression of these genes significantly increased in deprenyl-treated cells compared with that of undifferentiated cells; C. data were statistically significant at *p<0.05 as compared to control using one-way ANOVA. The mRNA expression of these genes significantly increased in deprenyl-treated cells compared with undifferentiated cells.

Before transplantation, we sought to determine if deprenyl-treated cells that were to be used as grafts truly included DA neurons. As shown in figure 2, there was no mRNA expression of TH and a weak band of Nurr1 in untreated cells, but the mRNA expression of these genes significantly increased in deprenyl-treated cells compared to undifferentiated cells.

The detectable level of DA released into supernatant was 8.9 ± 0.02 ng in treated cell.

### Apomorphine-induced rotation analysis

**Fig 3:**
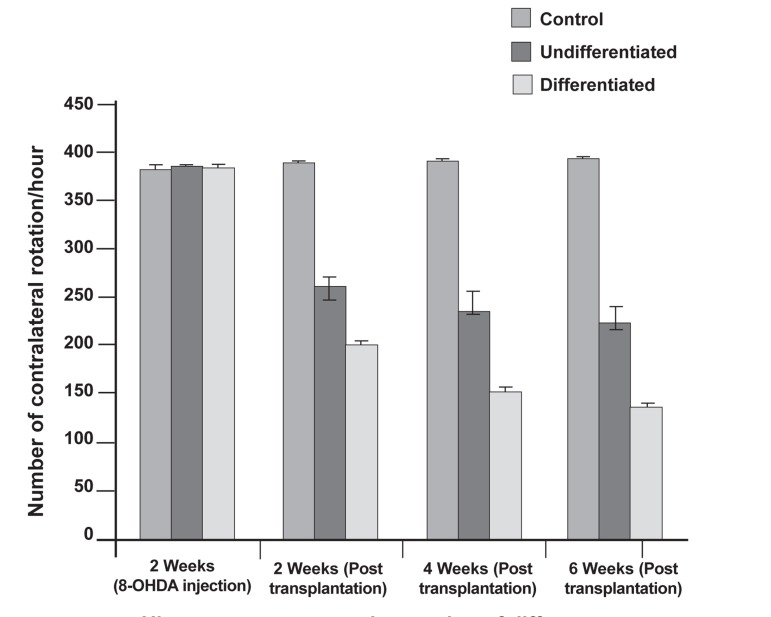
Analysis of rotational behavior induced by apomorphine. Apomorphine-induced circling behavior was evaluated at 2, 4, and 6 weeks after transplantation. Rats from the grafted groups were given BrdU-labeled bone marrow mesenchymal stem cells (BMSCs) that were undifferentiated and BrdU-labeled deprenyl- treated cells which were differentiated. Control animals received intrastriatal injections of medium. No recovery of rotational behavior was detected in control rats, whereas the grafted rats exhibited significantly decreased rotational behavior.

In figure 3 (2, 4, and 6 weeks after transplantation) the number of apomorphine-induced rotations significantly reduced in groups 2 and 3 compared to the control group (p≤0.05). There was a significant reduction of contralateral rotations to the lesion side in group 3 between 2 and 4 weeks, as well as between 2 and 6 weeks after transplantation, but no significant reduction in group 2 was observed during these two time periods.

The deprenyl-treated cell grafted animals had more rapid improvement of rotational behavior than BMSC-grafted animals.

### Histological and immunohistochemical analysis

**Fig 4:**
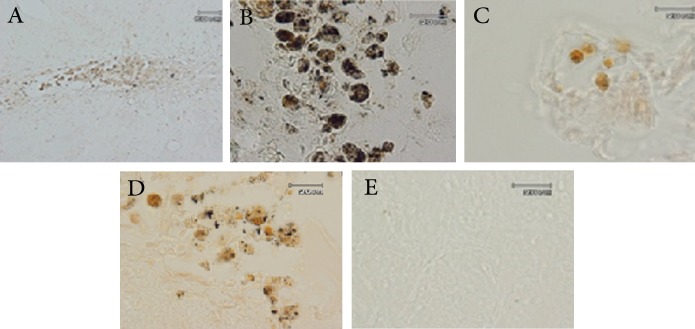
A. BrdU ^+^ cells at the implantation site; B. BrdU + transplanted cells are engrafted in the site of injury; C, D. cryosection of implantation site shows tyrosine hydroxylase- positive (TH +) cells; E. negative control (primary antibody was deleted).

Tissue sections were immunostained with anti-BrdU and anti-TH at the implantation site six weeks after grafting. BrdU^+^ or TH^+^ cells were observed in the striatum exclusively close to the grafted region, as illustrated in figure 4 A-E.

**Fig 5:**
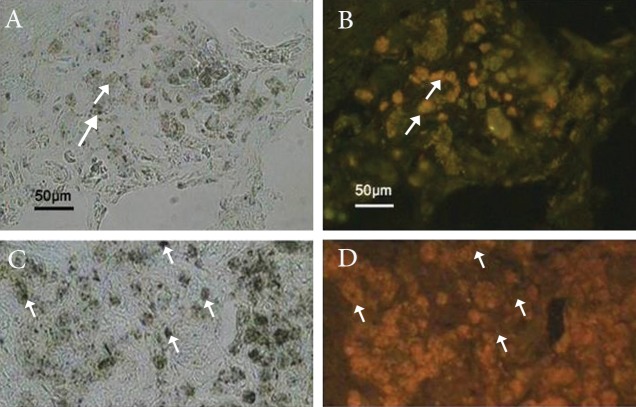
Immunostaining of the host striatum grafted with deprenyl-treated cells six weeks after transplantation. A, B, C, D are out of order. Please fix.) B. The tissue section was double-labeled with anti-neurofilament 200 antibody (secondary antibody conjugated with FITC) and anti-BrdU antibody (secondary antibody conjugated with rhodamine), then the two images were merged. A. Phase contrast photomicrograph of the field in the upper panel. D Upper panel: the tissue section was double labeled with anti-tyrosine hydroxylase (TH) antibody (secondary antibody conjugated with FITC) and anti-BrdU antibody (secondary antibody conjugated with rhodamine), then the two images were merged. C. Phase contrast photomicrograph of the field in the upper panel.

To ensure that transplanted cells that expressed neuronal lineage markers were of deprenyl-treated cell-origin, some sections were double stained for both a neuronal marker (NF200 or TH) and BrdU (Fig 5).

The majority of implanted areas were found to also express TH marker; the majority of grafted areas were aggregated with immunopositive cells. Therefore, we were unable to determine the number of TH^+^ cells present in the grafts.

Some of cells that were immunopositive for TH were also positive for BrdU, which demonstrated the presence of TH^+^ neurons derived from the transplanted cells had engrafted at the injury site.

### Electron microscopic study

**Fig 6:**
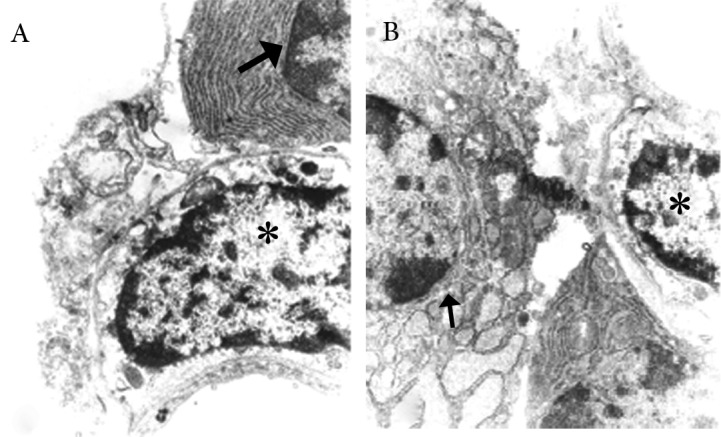
Electron microscopic study . A. Numerous amounts of polysomes and rough endoplasmic reticuli indicated immature neurons (arrow) at the implantation region; B. an astrocyte-like cell among the transplanted cells (shown by star).

The ultramicrostructure of the corpus striatum in figure 6 showed that in group 3 a few cells at the implantation region showed characteristics of immature neurons, which consisted of a large amount of polysomes and rough endoplasmic reticuli. Astrocyte-like cells were observed among the neurons at the implantation region.

## Discussion

Cell replacement therapy for PD is dependent upon a reliable source of purified DAergic neurons and the identification of factors relevant to their survival (2). The number of DAergic neurons that survive in the graft is relevant to the *in vitro* methods used to purify and quantify these cells prior to transplantation. DAergic neurons are derived from the fetal midbrain, embryonic, or adult stem cells. There are very low numbers of DAergic neurons in the fetal midbrain (depending on the dissected section), and more than 95-99% of cells are not DA neurons. Transplantation of these unwanted cells produces serious side effects, and in rare cases, even death

MSCs of the bone marrow are capable of differentiating along multiple lineages including neural cells, and have significant expansion capacity unlike other stem cells. BMSCs are capable of migrating to repair injured tissues (4). We have used BMSCs due to the numerous advantages for their clinical application. First, bone marrow is far more accessible than NSCs and ES cells. In addition, there are no ethical and immunological problems associated with BMSCs because they can be obtained from the patients, themselves. LU et al. (16) initially noted that BMSCs express not only mesodermal-related genes, but also endodermal and neuroectodermal-related genes even before neuronal induction, and therefore defined BMSCs not as "undifferentiated" but rather as "multidifferentiated" cells. Marrow stromal cell expression of several neural genes, even before induction, has been confirmed by several studies (34). Several in vitro culturing methods have thus far been developed to generate DAergic neurons from BMSCs. BMSCs can be considered a suitable candidate to undergo directed to a DAergic fate *in vitro* and as a cell source for autograft therapy of PD. BMSCs are the subject of treatment of neurodegenerative diseases by secreting neurotrophic factors. BMSCs could be directed to a DAergic fate by adding neurotrophic factors in a culture medium or by ectopic expression of their coding sequences within the cells. However, many of these chemicals have been reported to have mutagenic, teratogenic, or carcinogenic properties (15-17). Esmaeili et al. (35) has reported that deprenyl can induce ESCs differentiation into a neuronal phenotype with immunoreactivity to synaptophysin and TH. Ghorbanian et al. (19) showed that induced BMSCs were immunoreactive for NF 200, NF68, and synapsin I and expressed BDNF, NGF, NT-3, neuroligin 1, and PSD-95.

In this study, by using a specific differentiation protocol which was reported previously, we have demonstrated that it is possible to isolate an approximately pure population of DAergic neurons from BMSCs by deprenyl induction. Before transplantation, we examined whether the treated cells that were to be used as a graft actually had DAergic neurons. The results revealed that deprenyl could induce BMSCs differentiation into neuronal phenotype with synaptophysin, TH, NF200 immunoreactivity, dopamine secretion, and TH and Nurr1 gene expression. Deprenyl also had a trophic-like effect on cultured cells and stimulated their proliferation rate.

Nurr1 is involved in DAergic neuron development and survival, while the TH gene is related to DA synthesis (2). Analysis of semi-quantitative RT-PCR data revealed that the expression of Nurr1 and TH mRNAs of treated cells increased significantly compared with those of untreated cells. Next, we examined whether deprenyl-treated cells could survive and ameliorate some of the behavioral deficits after transplantation.

It was reported previously that rat BMSCs transplanted to 6-OHDA-lesioned rats differentiated into TH^+^ neurons. Transplanted mesenchymal cells appear to indirectly affect brain repair. Suggested mechanisms include production of growth factors, cytokines, and neurotrophic factors; promotion of the proliferation of endogenous progenitor cells; and the generation of favorable substrate for axonal growth or effects on the vasculature (36).

Apomorphine-induced rotation analysis showed improved rotational behavior in grafted animals compared to lesioned animals without any grafts. DAergic neuron transplantation caused behavioral recovery, which showed that intrastriatal DAergic grafts survive and establish synaptic connections with the host striatum and secrete dopamine.

Deprenyl-treated cell grafted animals showed more rapid improvement of rotational behavior than BMSCs-grafted animals. We observed a significant difference of motor behavior after injection of apomorphine, which was accompanied by the integration of TH^+^ cells at the lesioned side when compared with control (lesioned, medium injected) rats. Functional recovery required complete neural differentiation and integration with the host nervous system.

Six weeks after transplantation, grafted cells were immunohistochemically distinct from the host brain by positive immunoresponse; it seemed that donor cells were more fully integrated into the host tissue. To ensure that cells which expressed neuronal markers at the implantation site were of BMSCs origin, they were double-stained for both neuronal markers (NF-200 or TH) and BrdU. By double staining TH and BrdU, TH-positive neurons were identified at the implantation site, and we observed that transplanted cells survived and became well integrated into the host tissue.

## Conclusion

DAergic neurons that were produced in our differentiation protocol survived at the implantation area and reduced the lesion-induced circling behavior in a PD rat model. In addition, the striatum is a target area for DAergic neurons, and it may produce growth factor that increases the survival of DAergic neurons. DAergic neurons obtained from BMSCs in the presence of deprenyl expressed specific markers such as Nurr1 and TH, and released DA *in vitro*, were found to improve the behavioral deficits in Parkinsonian rat models.

In the future, we intend to identify factors, cells and conditions that will enhance the survival of deprenyl-induced TH^+^ neurons *in vivo* and *in vitro*. Our goal will be to identify essential growth factors relevant to DAergic neuron survival and proliferation rate; factors such as GDNF and the GDNF family members (neurturin, artemin, persephin), which are believed to have a neurotrophic effect on DAergic neurons.

This study may create the necessary foundation to generate consistently successful grafts in patients diagnosed with PD.
